# Primary Lung Adenocarcinoma Presenting with Pericardial Tamponade in a Young Adult: A Rare Case Presentation

**DOI:** 10.7759/cureus.6827

**Published:** 2020-01-31

**Authors:** Ivan Richard, Bracha Robinson, Giuseppe Filice, Jeffery Miskoff

**Affiliations:** 1 Internal Medicine, Hackensack Meridian Ocean Medical Center, Brick, USA

**Keywords:** pericardial tamponade, adeno carcinoma lung, young adults

## Abstract

Primary lung cancer typically presents in the older adult with a history of significant tobacco use. We present a young adult male with a rapid onset of dyspnea, and a large pleural and pericardial effusion with tamponade, necessitating urgent surgical intervention.

## Introduction

When a young male patient, in the third decade of life, presents with a lung mass and large pleural and pericardial effusion, amongst the most common pathologies initially considered are lymphoma, sarcoma, testicular carcinoma, and primary lung carcinoma. A review of the literature revealed about 2-5% of all lung cancer cases involve patients with an average age of 34 years, equal rates in men and women, and adenocarcinoma is the most common type of lung cancer [1,2]. A careful review of the past medical history, initial presentation, and physical exam findings are important to determine the initial workup and treatment. Regardless of the ultimate diagnosis, the initial management often involves critical care and surgical intervention rather than oncologic regimens. Once the patient is stabilized, a more long-term strategic treatment plan may be devised.

## Case presentation

A 34-year-old Caucasian man presented to the emergency room in August 2019 with progressive dyspnea of approximately one-week duration and was associated with worsening lower extremity swelling. He had no significant past medical history and was taking no prescription medications. He denied illicit drug use, but admitted to occasional marijuana use in the form of vaping. He also admitted to stopping alcohol approximately five years ago. He was otherwise never a smoker of tobacco. There was no family history of malignancy, bleeding, or thrombotic disorders. On physical examination, his vital signs were: blood pressure (BP) 112/72 mmHg, pulse rate (PR) 110 bpm, respiratory rate (RR) 24 rpm, peripheral capillary oxygen saturation (SpO2) 98% on 10 liters of a nonrebreather mask, and he was afebrile. He was in acute respiratory distress, speaking in short sentences. Jugular venous distention was 2 cm and there was left cervical chain lymphadenopathy. Breath sounds were absent in the right hemithorax but present on the left. Heart sounds were muffled, tachycardic, with a regular rhythm. His abdomen was mildly distended, nontender, with a palpable spleen and a nonpalpable liver. The patient had 1+ edema bilaterally in the lower extremities, which were warm and well perfused with 2+ peripheral pulses bilaterally. There were no focal neurological deficits found on physical exam. Testicular exam was normal.

**Figure 1:**
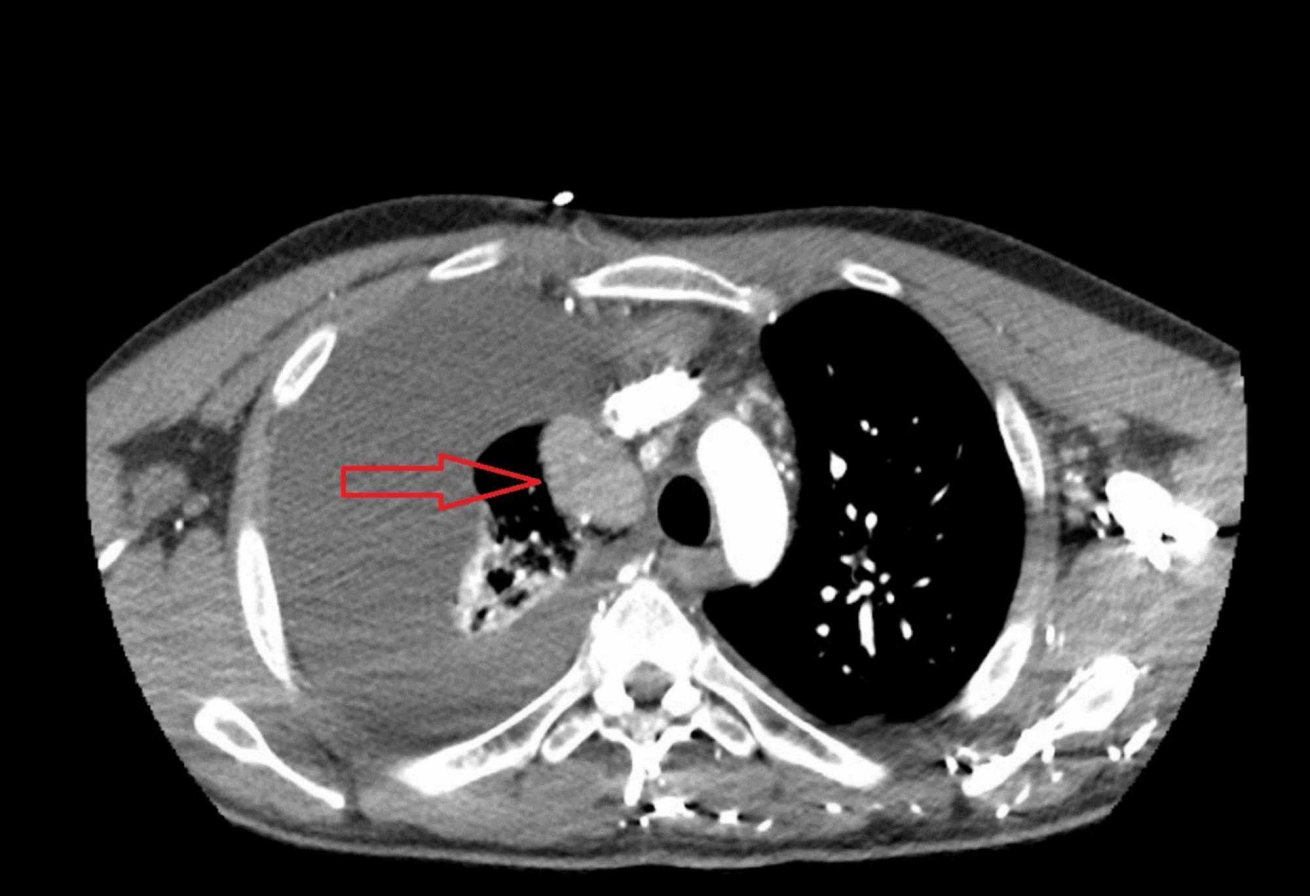
CT scan of the chest, transverse view, illustrating a right upper lobe mass (red arrow).

**Figure 2:**
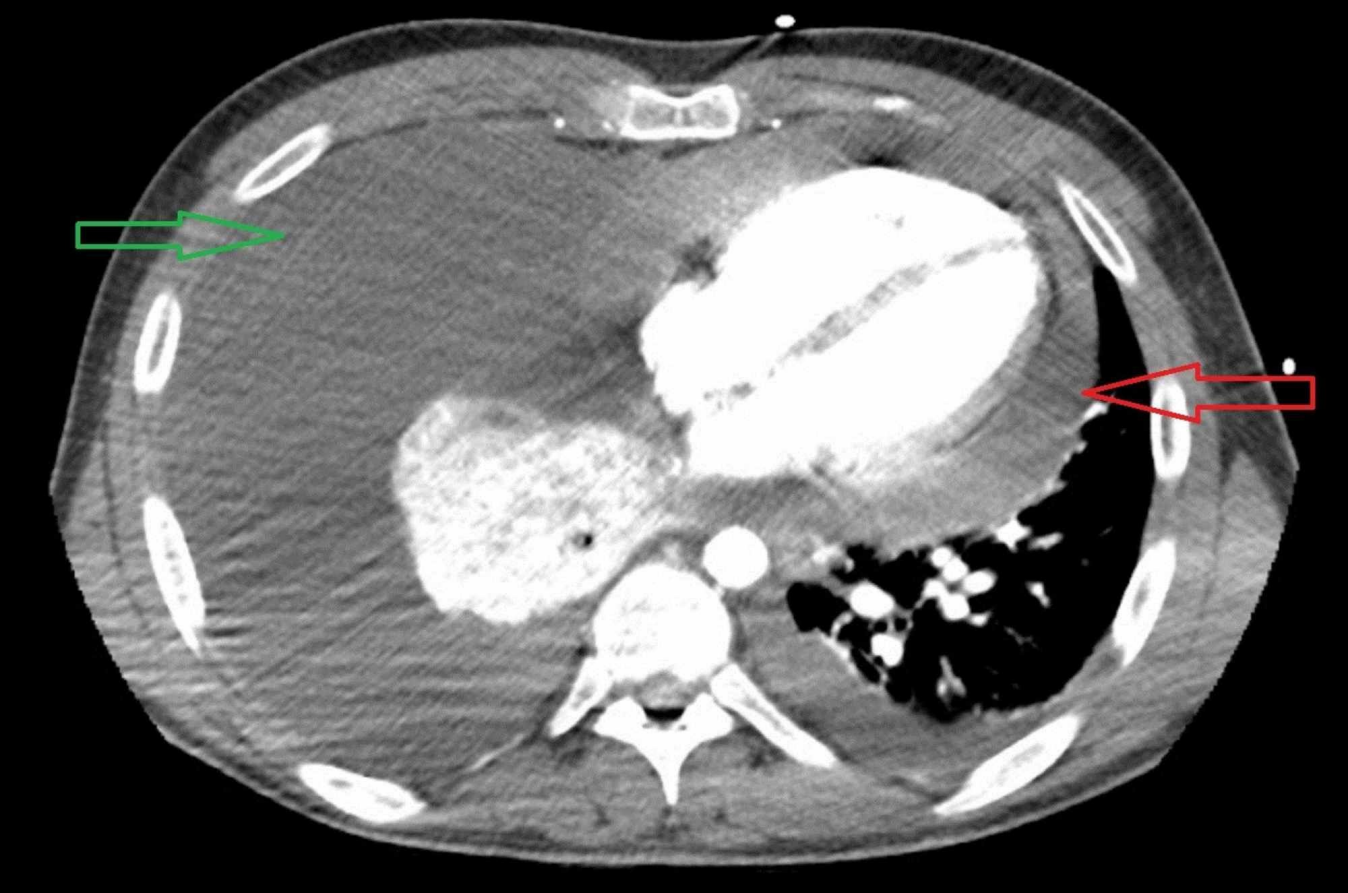
CT scan of the chest, transverse view, illustrating a pericardial tamponade (red arrow), and a large right pleural effusion causing atelectasis (green arrow).

**Figure 3:**
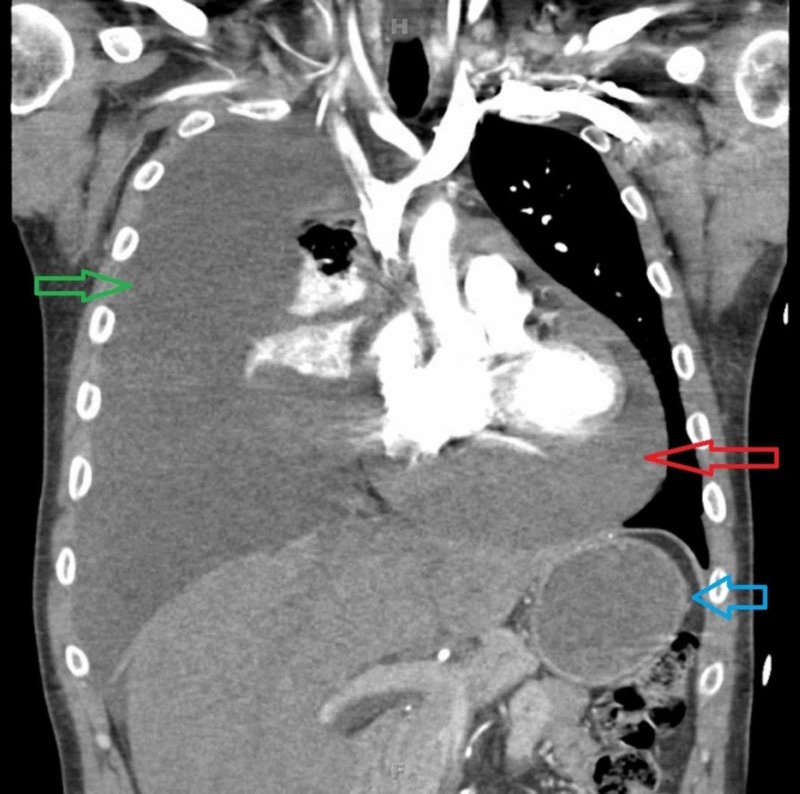
CT scan of the chest, coronal view, illustrating a pericardial tamponade (red arrow), a large right pleural effusion causing atelectasis (green arrow), and splenomegaly (blue arrow).

**Figure 4:**
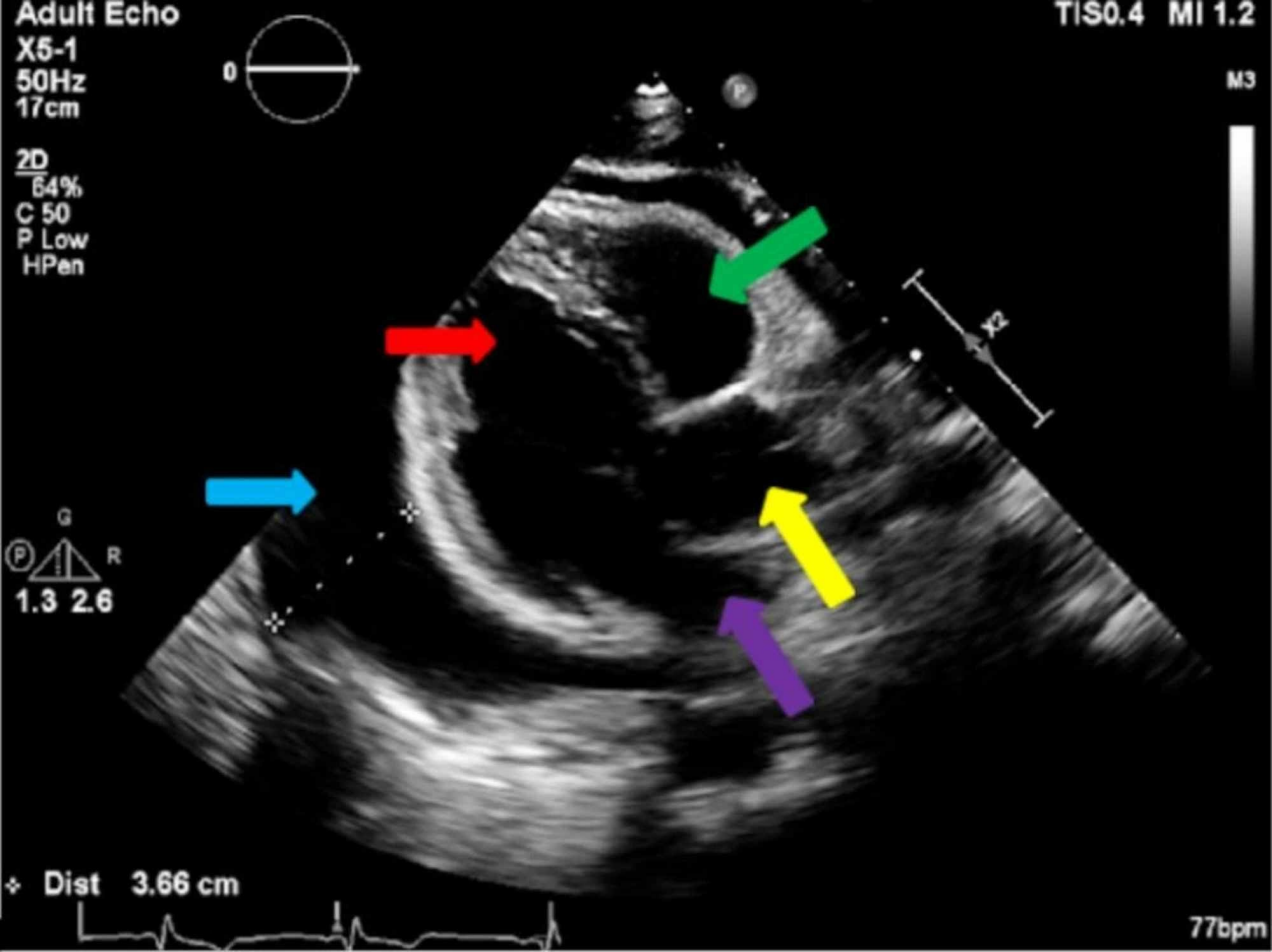
Parasternal long axis view of a large pericardial effusion measuring 3.66 cm (blue arrow), left ventricle (red arrow), compressed right ventricle (green arrow), aorta (yellow arrow), and the left atrium (purple arrow).

**Figure 5:**
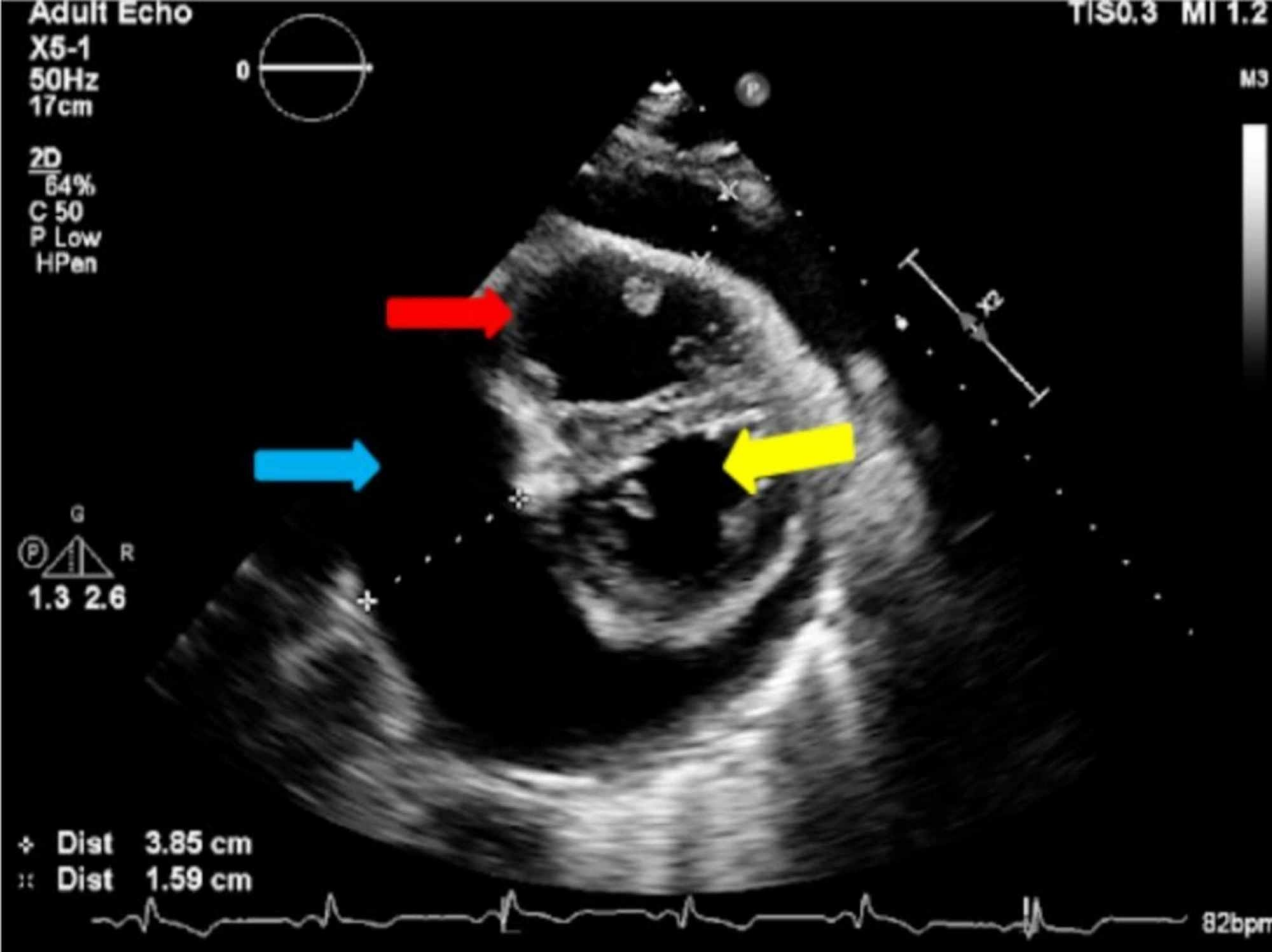
Parasternal short axis view of a large pericardial effusion measuring 3.85 cm (blue arrow), right ventricle (red arrow), and left ventricle (yellow arrow).

**Figure 6:**
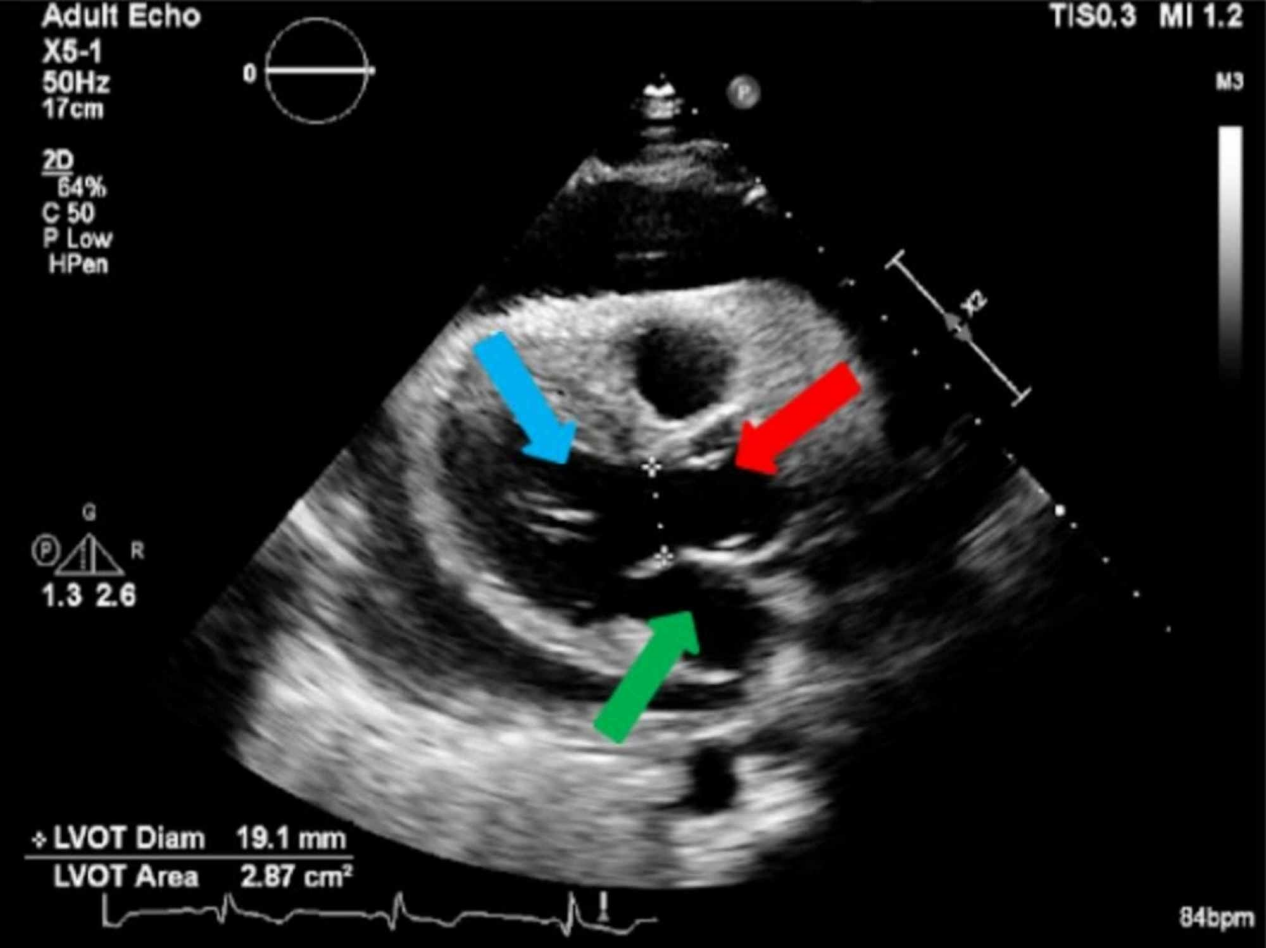
Parasternal long axis view of the left ventricle (blue arrow), aorta (red arrow), and the left atrium (green arrow).

**Figure 7:**
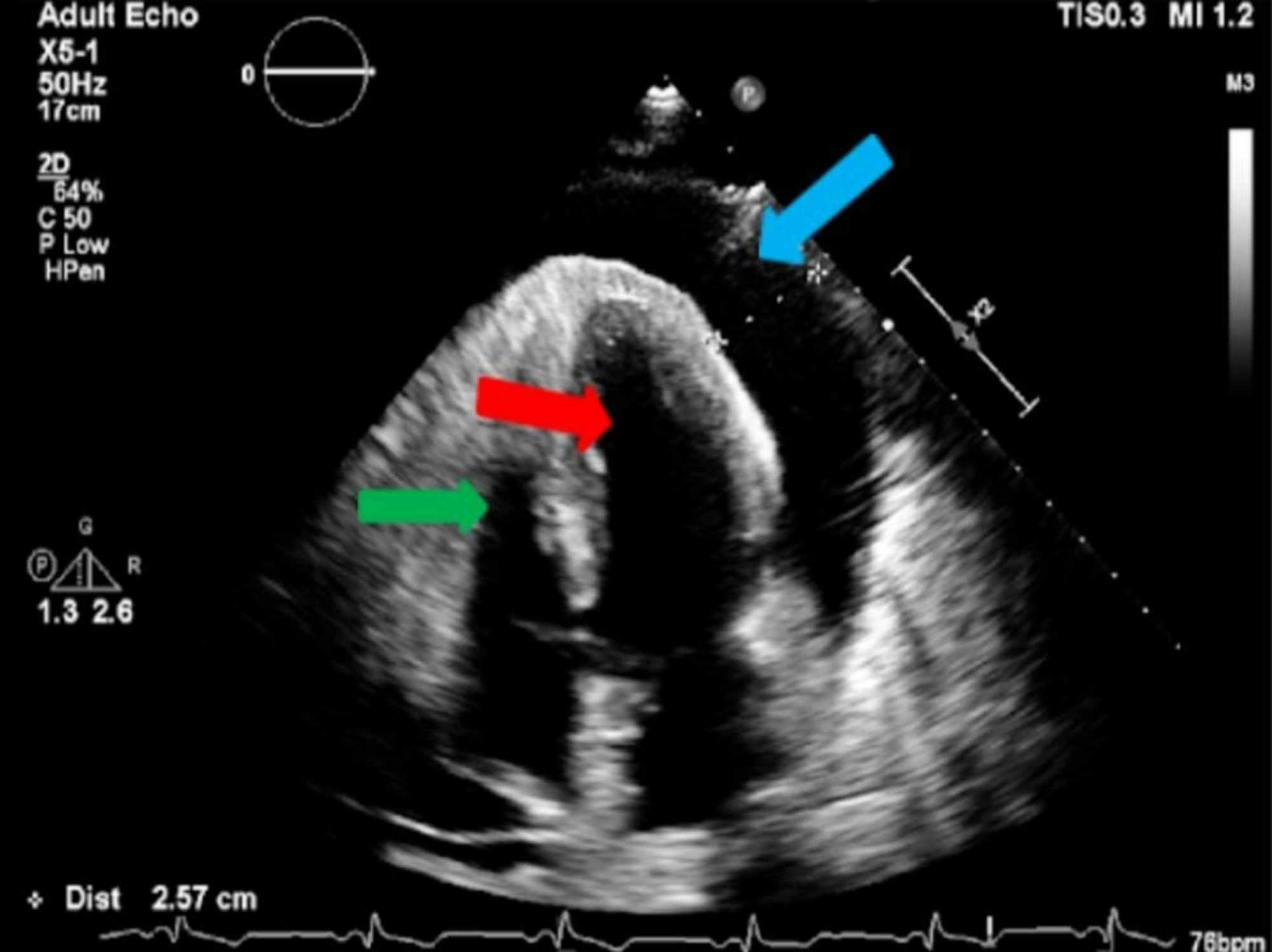
Apical 4 chamber view of a large pericardial effusion measuring 2.57 cm (blue arrow), left ventricle (red arrow), and right ventricle (green arrow).

Abnormal results of initial laboratory tests were as follows: creatinine 1.26 mg/dL (ref. interval 0.61-1.24 mg/dL), international normalized ratio (INR) 1.63 (ref. interval 0.88-1.15 ), D-dimer 871 ng/ml (ref. interval <501 ng/ml fibronectin equivalent units (FEU)). An ultrasound Doppler of bilateral lower extremities rule out deep vein thrombosis. While in the ultrasound department, the patient had a brief tonic-clonic seizure. CT of the head was negative. CT angiogram of chest found a large right pleural effusion with total compression atelectasis of the right lung, right upper lobe mass measuring 4 x 2.9 cm, several left lower lobe nodules, mediastinal and hilar lymphadenopathy, and a pericardial effusion (Figures 1-3). An echocardiogram showed early signs of pericardial tamponade (Figures 4-7). The patient was then admitted to the medical intensive care unit (MICU) in preparation for surgery. The cardiothoracic surgery service was consulted and took the patient to the operating room for a pericardial window with pericardial chest tube placement which drained 1.2 liters of hemorrhagic fluid and a right-sided chest tube which drained 3.7 liters. Samples of the pleural fluid and pericardial tissue were sent for cytology and pathology. At this point, the differential diagnosis was adenocarcinoma of lung vs lymphoma vs sarcoma vs testicular cancer.

The patient returned to MICU from post-anesthesia care unit (PACU) after successful pericardial window, awake and alert, but had an oxygen saturation of 88% and so was placed on optiflow. His saturation did not improve on optiflow and he developed hypotension and tachycardia. He was successfully intubated and sedated on Versed and fentanyl. Neosynephrine was ordered and the patient’s vital signs improved. On post-operation day (POD) 1, he spiked a fever with Tmax of 102 Fahrenheit (F), received Tylenol along with a cooling blanket, and blood cultures were obtained. Upon reviewing laboratory tests, the patient was found to have leukocytosis of 16.2 K/uL (ref. interval 4.5-11.0), lung infiltrates were detected on chest X-ray right, antibiotic therapy for pneumonia was initiated with vancomycin and aztreonam. The patient also developed hypotension of 85/46 mmHg despite neosynephrine at max dosage, therefore a central line was placed, fluid boluses of normal saline were given, and the patient was started on levophed. On POD 5, blood/sputum/acid fast/fungal/pericardial and pleural fluid culture resulted negative, serology IgG/IgM/IgA all resulted negative, and carcinoembryonic antigen (CEA)/alpha-fetoprotein (AFP)/hepatitis B and C (HEP B&C)/human immunodeficiency virus (HIV) were also negative. The patient’s electroencephalogram (EEG) report showed no epileptiform activity or focal pathology (ref. interval 0.88-1.15).

On POD 6, pathology samples resulted as negative but the cytology samples demonstrated malignant cells positive for AE1/AE3, MOC-31, TTF-1, and weakly for BerEP4 indicating pulmonary adenocarcinoma, stage 4 given the pericardial tamponade. The cytology specimens collected were not sufficient to run the required genetic markers. On POD 10, a video-assisted thoracoscopic surgery (VATS) biopsy of the right upper lobe lung mass confirmed invasive adenocarcinoma with predominant papillary and micro papillary features, moderately differentiated, pT2 Nx Mx. On POD 11, the patient was weaned off sedation, weaned off the ventilator and extubated. Post extubation he was awake, alert, and oriented to person, place, and time. A repeated echocardiogram found normal LV systolic function and no effusion. A magnetic resonance imaging (MRI) of the brain and a bone scan found no signs of metastasis. On discharge POD 24, the patient was advised to follow up with oncology for outpatient positron emission tomography (PET) scan and treatment with Keytruda.

## Discussion

**Figure 8:**
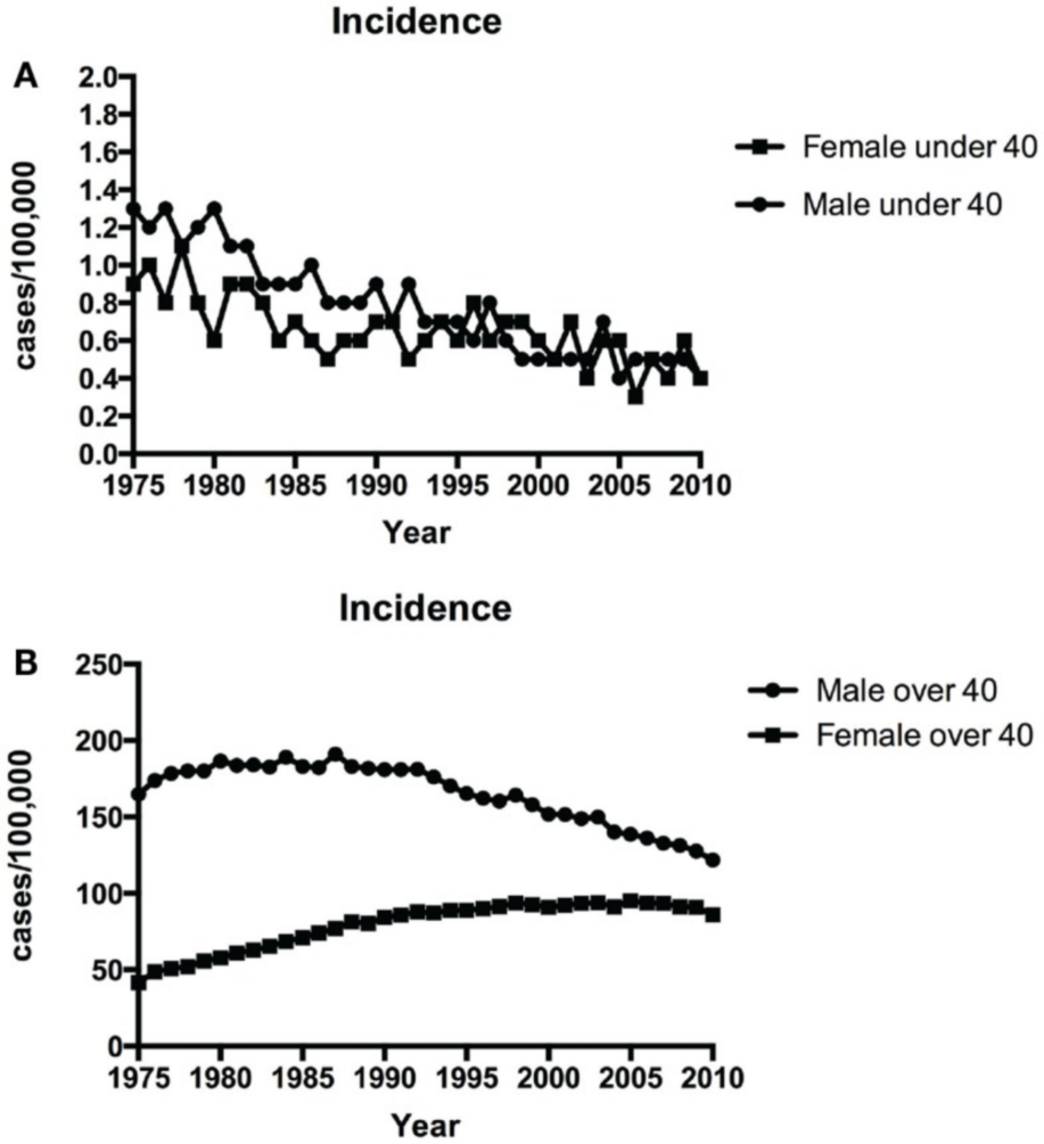
Charts A & B illustrate the number of NSCLC presenting as primary cancer in those under 40 years old (Chart A) and in those over 40 years old (Chart B). NSCLC: Non-small cell lung carcinoma [2]

The mainstay of treatment for pericardial tamponade in the setting of non-small cell lung carcinoma (NSCLC) is pericardial window. After which the average survival is three months or less. Such a poor prognosis relates to the advanced stage of lung cancer in these patients. In 4%-7% of cases, acute pericardial disease is a sign of an undiscovered malignancy, usually a primary lung cancer [3]. In a young male without any recent cardiac procedures or history of coronary artery disease or renal failure, other possibilities such as hemorrhagic effusion should be considered, in addition to metastatic cancer, including testicular cancer and lymphoma, autoimmune disorders such as systemic lupus erythematosus, and infectious causes such as tuberculosis and HIV (Figure 8) [4]. Pleural and pericardial effusions when found together are associated with malignancy more often than pericardial effusions alone [5-6]. In the past decade, studies have shown that systemic chemotherapy with pericardial window is more effective than systemic chemotherapy or systemic chemotherapy with drainage [7-9]. Other recent studies have found local chemotherapy with or without systemic chemotherapy in addition to pericardiocentesis is superior to other treatment options [5,7]. The first case series that followed patients on modern chemotherapy and molecular targeted therapy found the median survival was 4.5 months, with two patients alive at 15 and 17 months [10]. We hope that our case report sheds light on the need for more clinical trials on chemotherapy either systemic or local in addition to pericardial window as treatment for malignant pericardial effusions/tamponade focusing on improving length of survival.

## Conclusions

We described a patient in his third decade of life presenting with NSCLC, and commonly this type of cancer often produces mild or no symptoms until the disease is well advanced; early diagnosis may be helpful to improve outcomes. A careful physical exam, imaging, surgical intervention and critical care management were crucial in this case. Initially, based on the patient’s relatively young age, the hope was that he would have a curable disease, or one with at least a long-term survival. Ultimately, a diagnosis was made of the most common type of lung carcinoma in the adult population. No obvious identifiable risk factors were uncovered, however, lung cancer screening of other primary relatives may be indicated based on current guidelines. Future clinical trials will likely lead to updated guidelines and recommendations in addition to improved treatments with improved outcomes.
